# Supplementary material for: COVID-19 epidemiology and changes in health service utilization in Azraq and Zaatari refugee camps in Jordan: A retrospective cohort study
Source: PLoS Med. 2022 May 10;19(5):e1003993. doi: 10.1371/journal.pmed.1003993 (PMC9089859; doi:10.1371/journal.pmed.1003993)
Supplement: S2 Supporting Information — Table A: Definition of outcome indicators included in the interrupted time series analysis. Table B: Model specification for interrupted time series analysis. (DOCX) [file pmed.1003993.s002.docx]

**S2 – Additional details related to the study methods**

**Table of Contents**

[1 Definition of outcome indicators included in the analysis 1](#_Toc101520196)

[2 Definitions of respiratory infections as reported in UNHCR’s Jordan HIS 2](#_Toc101520197)

[3 Interrupted Time Series – model specifications 2](#_Toc101520198)

[4 Analysis Plan 3](#_Toc101520199)

# Definition of outcome indicators included in the analysis

Table A: Definition of outcome indicators included in the Interrupted Time Series analysis.

| **Indicator name** | **Definition** | **Numerator** | **Denominator** |
| --- | --- | --- | --- |
| Health utilization rate | Average number of consultations per person per year | Number of consultations per month multiplied by 12 | Population |
| ANC1 coverage | Coverage of antenatal care | Number of first antenatal consultation per month | Number of pregnant women was calculated by multiplying the population size / 1,000 by the quarterly crude birth rate (births / 1,000 people / month) inflated by expected percentage of miscarriages and abortions (12.5%) |
| Coverage of live births attended by medical and paramedical personnel | Proportion of births attended by medical and paramedical personnel | Number of live births attended by medical and paramedical personnel | Expected number of monthly deliveries, estimated by multiplying total camp population by the crude birth rate |
| Family planning consultation rate |  | Number of new and repeat consultations for Family Planning per month multiplied by 1,000 by 12 | Estimated population |
| New FP consultations |  | Number of new consultations for Family Planning per month multiplied by 1,000 by 12 | Estimated population |
| Vaccination coverage Measles | Proportion of children 9 to 59 months vaccinated with MCV | Number of children (9-59 months of age) who received MCV per month multiplied by 12 | Estimated children population 9 to 59 months (all children between 13 and 59 months plus 20% of the population under 12 months) |
| Annual rate of respiratory infections (by type of infection: lower, upper respiratory infections, influenza like illness) | Average number of consultations (by type of respiratory infections) per year per 1,000 | Number of consultations for respiratory infections by type per month multiplied by 1,000 by 12 | Estimated population |
| Crude annual rate of respiratory infections |  | Number of LRTI + URTI + ILI consultations multiplied by 1,000 by 12 | Estimated population |
| Consultation rate for diabetes |  | Number of diabetes consultations per month multiplied by 1,000 by 12 | Estimated population |
| Consultation rate for injuries |  | Number of consultations for injuries per month multiplied by 1,000 by 12 | Estimated population |
| Crude mortality rate | Number of deaths per 1,000 in the total population per year | Number of deaths all causes, all ages per month multiplied by 1,000 by 12 | Estimated population |

# Definitions of respiratory infections as reported in UNHCR’s Jordan HIS

UNHCR’s HIS differentiate between three types of respiratory infections:

- Lower tract respiratory infections (LRTI), which include
  - Adults (5 years or older) with temperature higher or equal to 38C or subjective fever, and cough or sore throat; and breathing rate > 200 breaths/minute.
  - Children (2months to 5 years of age) with cough or difficulty breathing; and any one of the following general danger signs: breathing rate > 50 breaths/minute (for infant 2 to 12 months) or breathing rate > 40 breaths/minute (for children 1 to 5 years); chest indrawing; stridor in a calm child; unable to drink or breastfeed; vomit; convulsion, lethargic or unconscious.
  - Infants (1 week to 2 months) with any one of the following: breathing > 60 breaths / minute; severe chest indrawing; nasal flaring; grunting.
- Upper tract respiratory infections (URTI), which include patients presenting with cough/cold (non pneumonia, and running nose, cough and low grade fever.
- Influenza Like Illness (ILI), which are defined as adults or children with measured temperature > 38C, and cough with onset within the last 10 days.

# Interrupted Time Series – model specifications

Table B: Model specification for Interrupted Time Series analysis.

|  | **Model specification** | | |
| --- | --- | --- | --- |
|  | Core predictors | Seasonality | Other modifications to the model |
| Health utilization rate | Yes | Included | No |
| Mortality | Yes | Removed | i) Tested for lag, but no lag introduced;  ii) mortality spikes noticed 2018 (Q1+Q2) 🡪 removed to improve model fit |
| LRTI | Yes | Included | No |
| URTI | Yes | Included | No |
| ILI | Yes | Included | Gaussian model (link =log) was used to improve model fit with NB |
| all RTI | Yes | Included | No |
| Diarrhea | Yes | Included | Gaussian model (link=log) was used |
| Diabetes | Yes | Removed | No |
| Injury | Yes | Included | No |
| FP | Yes | Removed | No |
| new FP only | Yes | Removed | No |
| ANC1 coverage | Yes | Removed | No |
| Live births coverage | Yes | Removed | No |
| Measles vaccination coverage | Yes | Removed | Abnormal spikes (likely due to vaccination campaigns removed) |
| SAM | Yes | Removed | Abnormal spikes (likely due to mass screening removed) |

Note: Core predictors include population; Centered Month_i­_; COVID; Month since COVID_i_

# Analysis Plan

May 3, 2021

**The Health Impacts of COVID-19 on Refugees:**

**COVID-19 and non-COVID-19 data analysis plan**

A Collaboration between JHU and UNHCR under the CDC Cooperative Agreement

1. **Objectives**

This study aims to investigate the direct and indirect effects of COVID-19 in two refugee settings to:

1. Better understand the epidemiology of SARS-CoV-2 infections among refugee populations; and
2. Examine how non-COVID-related health services and health outcomes have been affected by the pandemic.

COVID and non-COVID-related data will be compared with the specific policies and public health measures implemented by each country and setting to prepare and respond to the epidemic.

The research sites where the study will be implemented include Uganda (the settlements in the north, as well as refugees in Kampala) and Jordan (both refugee camps of Zaatari and Azraq as well as refugees in Amman). Inclusion of urban refugees will depend on data availability.

1. **Research questions**
   1. What is the epidemiology of COVID-19 among refugee populations?
      1. Who are the most affected?
      2. Which clinical signs do COVID-19 cases in refugee settings present and what are the most common type of exposure?
      3. What are the outcomes of clinical management?
   2. How has the testing, contact tracing and vaccination capacity evolved since the beginning of the pandemic?
   3. How has the non-COVID-19-related morbidity and mortality changed over time since the beginning of the pandemic?
   4. How has COVID-19 affected other health services?
      1. How have consultations changed since the beginning of the pandemic?
      2. Could essential health services be continued?
      3. How has the provision of health services been adapted?
2. **Data**

Following data sources will be utilized in this analysis:

COVID-19-related data

- Line list of COVID-19 data per camp and country
- List of people tested (if available) or aggregated data on COVID-19 testing conducted
- Data on vaccination implementation

Routine health services

- Monthly data from UNHCR Health Information System (HIS)

Policies and public health measures

- National policies and implementation strategies on COVID-19 prevention, testing, and vaccination
- UNHCR and partners’ adaptation protocols

1. **Overall analysis approach**

COVID-19 data

The analysis will encompass following topics:

- Testing
- General case information
- Clinical characteristics
- Exposure
- Case management
- Contact tracing
- Vaccination

As a general approach, following steps will be taken:

- Descriptive statistics of counts, frequency, proportions
- Rates (for example, incidence rate, vaccination rate, testing rate)
- Probability of a given outcome
- Association with selected covariables

Comparisons between camps/settlements, as well as with host country and with urban areas will be conducted depending on data availability. Evolution over time will be investigated against implemented policies and measures.

Routine Health data

The analysis will encompass following topics:

- General consultation
- Maternal health (antenatal, delivery, postnatal care)
- Child health (vaccination and nutrition)
- HIV and TB
- Service delivery (health care workers availability, bed capacity/utilization)
- Morbidity and mortality (crude and for selected diseases)

Trend analysis to investigate changes over time will be conducted. Disaggregated analysis to investigate whether specific population groups or specific services were most affected. Comparisons between camps/settlements, as well as with host country and with urban areas will be conducted depending on data availability. Evolution over time will be investigated against implemented policies and measures.

1. **List of indicators**
   1. **COVID-19 indicators by thematic area**

| **Indic #** | **Indicator** | **Numerator** | **Denominator** | **Disaggregation / explanatory variables** | **Source** |
| --- | --- | --- | --- | --- | --- |
| **1. Testing** | | | | | |
| 1.1 | # of tests per month |  |  | Location | Aggregated testing data |
| 1.2 | Testing rate (total) | # of tests conducted | Population /100,000 | sex, age, location | Aggregated testing data |
| 1.3 | Positivity rate (total) | # of positive tests | # of tests conducted | sex, age, location | Aggregated testing data |
| 1.4 | Testing rate over time | # of tests conducted per month | Population /100,000 |  | Aggregated testing data |
| 1.5 | Positivity rate over time | # of positive tests per month | # of tests conducted per month |  | Aggregated testing data |
| 1.6 | Proportion of cases symptomatic at time of specimen collection | # of COVID cases who are symptomatic at time of specimen collection | # of COVID specimen collections | age; sex | COVID line list |
| 1.7 | Proportion of cases symptomatic at time of specimen collection per month (evolution over time) | # of COVID cases who are symptomatic at time of specimen collection per month | # of COVID specimen collections per month |  | COVID line list |
| 1.8 | Proportion of reasons why the case was tested / types of surveillance | # of cases tested by reason | Total # of cases |  | COVID line list |
| 1.9 | Time btw symptoms onset and sample collection | Difference between date of specimen collection and date of symptom onset |  | sex | COVID line list |
| 1.10 | Time btw sample collection and results | Difference between date of results and date of specimen collection | NA | sex | COVID line list |
| **2. General cases info** | | | | | |
| 2.1 | Counts of cases and death and epidemiological curve |  |  | sex, age, location | COVID line list |
| 2.2 | Distribution of cases by age, sex, district/camp | # of cases (with a given characteristics) | Total # of cases | sex, age, location | COVID line list |
| 2.3 | Average age of cases | Sum of age of cases | total # of cases | sex, age, location | COVID line list |
| 2.4 | Incidence rate | # of positive cases per month | Population /100,000 | sex, age, location | COVID line list |
| **3. Clinical characteristics** | | | | | |
| 3.1 | Proportion of cases who are symptomatic | # of cases with symptoms at specimen collection | Total # of COVID cases | sex, age, location | COVID line list |
| 3.2 | Proportion of reported symptoms | # of cases by symptoms | # of cases reporting any symptoms |  |  |
| 3.3 | Odds ratio of being asymptomatic (by sex) | # of F COVID cases who are symptomatic / # of F COVID cases asymptomatic | # of M COVID cases who are symptomatic / # of M COVID cases asymptomatic | age, sex | COVID line list |
| 3.4 | Proportion of cases with comorbidities | # of cases with at least one comorbidity | Total # of cases | sex, age, location | COVID line list |
| 3.5 | Proportion of cases by number of comorbidities  3.5.1: 1 comorbidity  5.5.2: 2 or more comorbidities | # of cases with 1  # of cases with 2 or more comorbidities | Total number of cases  Total number of cases with comorbidities | age, sex | COVID line list |
| 3.6 | Proportion of cases by specific comorbidity | # of cases by comorbidity | Total # of cases with comorbidities | sex, age, location | COVID line list |
| **4. Exposure** | | | | | |
| 4.1 | Proportion of cases who are health care workers | # of health care workers among the cases | Total # of cases | sex, age, location | COVID line list |
| 4.2 | Incidence rate among health care workers (HCW) | # of cases who are health care workers in a month | # of health care workers in the camps / 100,000 | location | COVID line list + # of HCW by location |
| 4.3 | Proportion of cases who travelled outside the camp | # of cases who reported traveling outside the camp | Total # of cases | sex, age, location | COVID line list |
| 4.4 | Proportion of cases who visited a HF before symptom onset | # of cases who reported visiting a HF in the 14 days before symptom onset (or before testing if asymptomatic) | Total # of cases | sex, age, location | COVID line list |
| 4.5 | Proportion of cases who had contact with one or more other COVID cases | # of cases who reported having contact with other COVID cases | Total # of cases | sex, age, location | COVID line list |
| 4.6 | Proportion of cases who reported having contact with other COVID cases by contact setting^[[1]](#footnote-1)^ | # of cases who reported having contact with other COVID cases (by type of setting) | Total # of cases | sex, age, location | COVID line list |
| **5. Case management** | | | | | |
| 5.1 | Proportions of cases hospitalized | # of cases hospitalized | Total # of cases | sex, age, location | COVID line list |
| 5.2 | Proportions of cases isolated^[[2]](#footnote-2)^ | # of cases isolated | Total # of cases | sex, age, location | COVID line list |
| 5.3 | Proportions of cases ICU | # of cases in ICU | Total # of cases  Number of cases hospitalized | sex, age, location | COVID line list |
| 5.4 | Proportions of cases on ventilator | # of cases on ventilator | Total # of cases  Number of cases hospitalized | sex, age, location | COVID line list |
| 5.5 | Proportion of cases receiving oxygen^[[3]](#footnote-3)^ | # of cases on oxygen | Total # of cases  Number of cases hospitalized | sex, age, location | COVID line list |
| 5.6 | Time btw symptoms onset and hospitalization | Difference between date of hospitalization and of symptoms onset |  |  | COVID line list |
| 5.7 | Length of stay at the hospital | Difference between date of hospitalization and date of discharge |  | sex | COVID line list |
| 5.8 | Duration of isolation | Difference between date of isolation and date of discharge |  | sex | COVID line list |
| 5.9 | Bed occupancy^[[4]](#footnote-4)^ | # of beds occupied in the emergency ward | Total # of beds in the emergency ward |  | Health facility data |
| 5.10 | Probability of hospitalization (odds ratio by different risk factors as per column 4 – definition provided for sex) | # of F COVID cases who are hospitalized / # of F COVID cases non-hospitalized | # of M COVID cases who are hospitalized / # of M COVID cases non-hospitalized | age, sex, comorbidities, delay btw symptoms and tests, symptomatic  can be calculated for  i) Total # of cases or  ii) Number of cases hospitalized | COVID line list |
| 5.11 | Probability of isolation (odds ratio by different variables as per column 4 - definition provided for sex) | # of F COVID cases who are isolated / # of F COVID cases non-isolated | # of M COVID cases who are isolated / # of M COVID cases non-isolated |  | COVID line list |
| 5.12 | Probability of ICU (odds ratio by different risk factors as per column 4 - definition provided for sex) | # of F COVID cases who are in ICU / # of F COVID cases who are not in ICU | # of M COVID cases who are in ICU / # of M COVID cases not in ICU |  | COVID line list |
| 5.13 | Probability of ventilator (odds ratio by different risk factors as per column 4 - definition provided for sex) | # of F COVID cases who are on a ventilator / # of F COVID cases who are not on a ventilator | # of M COVID cases who are on a ventilator / # of M COVID cases who are not on a ventilator |  | COVID line list |
| 5.14 | Probability of death (odds ratio by different risk factors as per column 4 - definition provided for sex) | # of F COVID cases who died / # of F COVID cases who did not die | # of M COVID cases who died / # of M COVID cases who did not die |  | COVID line list |
| **6. Contact tracing** | | | | | |
| 6.1 | Average # of contacts listed per case | # of contacts listed per month | Total # of cases per month |  | Contact tracing data |
| 6.2 | % of contacts tested per month | # of contacts tested per month | # of contacts listed per month |  | Contact tracing data |
| 6.3 | % of contacts that are followed | # of contacts that are followed | # of contacts listed |  | Contact tracing data |
| 6.4 | Secondary attack rate | # of contacts tested positive | Total # of contacts |  | Contact tracing data |
| 6.5 | % of cases in isolation who were monitored for 14 days | # of cases in isolation monitored for 14 days | # of cases in isolation | location | Contact tracing data |
| 6.6 | % of contacts who develop symptoms | # of contacts who develop symptoms | # of contacts listed |  | Contact tracing data |
| **7. Vaccination** | | | | | |
| 7.1 | Vaccination rate (1st dose) | # of vaccine doses delivered per month 1st dose | Population / 100,000 | Location | Vaccination Data |
| 7.2 | Vaccination rate (2nd dose) (if applicable) | # of vaccine doses delivered per month 2nd dose | Population / 100,000 | Location | Vaccination Data |
| 7.3 | Proportion of the population who received at least one dose | Number of people who received at least one dose of COVID-19 vaccine | Total eligible population | Location | Vaccination data |
| 7.4 | Proportion of the population who is fully vaccinated | Number of people who have received all doses prescribed by the vaccination protocol | Total eligible population | Location | Vaccination data |

- 1. **Routine Health services**

| **Indic #** | **Indicator name** | **Numerator** | **Denominator** | **Disaggregation** | **Source** |
| --- | --- | --- | --- | --- | --- |
| **A.** | **Service Provision** |  |  |  |  |
| **1. Consultations** | | | | | |
| 1.1 | # of total outpatient consultations per month | # of total consultations for all conditions, all age groups |  | sex; age (> / < 5) | HIS |
| 1.2 | # of outpatient consultations for malaria per month | # of consultations for malaria (suspected and confirmed) |  | sex; age (> / < 5) | HIS |
| 1.3 | Proportion of malaria cases that are confirmed by RDT | # of malaria cases confirmed by RDT | # of total malaria cases (suspected and confirmed) | sex; age (> / < 5) | HIS |
| 1.4 | # of monthly outpatient consultations for upper respiratory infection (URI) | # of consultations for URI |  | sex; age (> / < 5) | HIS |
| 1.5 | # of monthly outpatient consultations for lower respiratory infections (LRI) | # of consultations for LRI |  | sex; age (> / < 5) | HIS |
| 1.6 | # of monthly outpatient consultations for diarrhea | # of consultations for diarrhea |  | sex; age (> / < 5) | HIS |
| 1.7 | # of monthly outpatient consultations for diabetes | # of consultations for diabetes |  | sex; age (> / < 5) | HIS |
| 1.8 | # of monthly outpatient consultations for injuries (trauma) | # of consultations for injuries |  | sex; age (> / < 5) | HIS |
| 1.9 | # of patients referred from the health facility to referral hospital | Total # of patients referred to the referral hospitals |  | sex; age (> / < 5) | HIS |
| 1.10 | # of emergency referrals from health facility to referral hospitals | # of emergency referrals from health facility to referral hospitals |  | sex; age (> / < 5) | HIS |
| 1.11 | # of consultations per health care worker per day | # of total consultations (all ages, all causes) per day | # of HCW by health facility |  | HIS + # of HCW |
| 1.12 | # of consultations per person per year | # of total consultations (all ages, all causes) | Population estimates |  | HIS + population data |

| **2. Health service delivery** | | | | | |
| --- | --- | --- | --- | --- | --- |
| 2.1 | # and trend of health care workers^[[5]](#footnote-5)^ | Tbc |  | tbc | HIS |
|  | Availability of essential medicines |  |  |  |  |
| 2.2 | # of days of stock out of antibiotics^[[6]](#footnote-6)^ per month | # of days per month with stock out of antibiotics | 30 |  | HIS |
| 2.3 | # of days of stock out of malaria treatment for children U5 (and /or adult)^[[7]](#footnote-7)^ per month | # of days per month with stock out of antimalarials for children | 30 |  | HIS |
| 2.4 | # of days of stock out of DPT vaccine doses per month | # of days per month with stock out of DPT vaccine | 30 |  | HIS |
| **3. Antenatal care** | | | | | |
| 3.1 | # of Antenatal Care consultations - first visit (per month) | # of women aged 15–49 who attended the first ANC visit with skilled health personnel |  |  | HIS |
| 3.2 | # of Antenatal Care consultations - fourth visit (per month) | # of women aged 15–49 who attended the fourth ANC visit with skilled health personnel |  |  | HIS |
| 3.3 | Coverage of ANC4 - Percent of women who attended ANC with skilled health personnel at least four times during pregnancy | # of women aged 15–49 who attended the fourth ANC visit with skilled health personnel | # of women ages 15–49 who attended the first ANC visit |  | HIS |
| **4. Labor and delivery** | | | | | |
| 4.1 | # of institutional deliveries per month | # of live births in a health facility per month |  |  | HIS |
| 4.2 | Proportion of facility-based deliveries | # of live births in a health facility | Estimated # of live births in population in a given period |  | HIS + population estimates |
| 4.3 | # of cesarian sections performed at hospitals per month | # of cesarian sections performed at hospitals per month |  |  | HIS |
| 4.4 | Proportion of cesarian sections | # of cesarian sections in a health facility | # of total deliveries in the health facility |  | HIS |
| **5. Postnatal care** | | | | | |
| 5.1 | # of Post-Natal Care consultations for women and newborn during the first 48 hours following birth per month | # of women and newborn who attended by health care provider during the first 48 hours following birth |  |  | HIS |
| 5.2 | Proportion of mothers and newborns attended by a health care provider during the first 48 hours following birth per month | # of women and newborn attended by health care provider during the first 48 hours following birth per month | Administrative estimate of the # of births in the reporting period in the catchment area |  | HIS + population estimates |
| **6. Family Planning** | | | | | |
| 6.1 | # of new and old consultations for family planning (per month). | # of new and old consultations for family planning. |  | new; old | HIS |
| 6.2 | % of new FP clients who adopt oral contraceptive pills, injectables, or long acting reversible methods (implants, IUD) or permanent methods (TL)  Or Proportion of modern methods distributed (out of all methods) | New FP clients at facilities who adopt oral contraceptive pills, injectables, or long acting reversible methods (implants, IUD) or permanent methods (TL)  Number of modern methods distributed | Total # of new FP clients  Total # of methods distributed |  | HIS |
| **7. Vaccination coverage^[[8]](#footnote-8)^ (Measles, DPT, Polio, BCG, Yellow Fever, Rotavirus)** | | | | | |
| 7.1 | # of vaccination doses provided to children < 12 months by vaccine (per month) |  |  |  | HIS |
| 7.2 | Vaccination coverage (DPT3, Polio, BCG, Yellow Fever, Rotavirus, Measles) | # of administered doses by vaccine among children < 12 month | Estimated # of infants in the given area |  | HIS |
| **8. Morbidity** | | | | | |
| 8.1 | Malaria^[[9]](#footnote-9)^ (trend of cases) per month | # of malaria cases, all ages |  | age (> / < 5) | HIS |
| 8.2 | Malaria (proportional morbidity) | # of malaria cases | # of total consultations for acute conditions | age (> / < 5) | HIS |
| 8.3 | Acute Respiratory infections (ARI) (trend of cases) per month | # of ARI cases |  | age (> / < 5) | HIS |
| 8.4 | 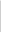Acute Respiratory infections (% morbidity) | # of consultations for ARI | # of total consultations for acute conditions | age (> / < 5) | HIS |
| 8.5 | Cholera (trend of cases) per month | # of cholera cases |  | age (> / < 5) | HIS |
| 8.6 | Bloody diarrhea per month | # of bloody diarrhea cases |  | age (> / < 5) | HIS |
| 8.7 | 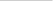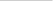Diarrhea / diarrheal diseases (% morbidity) | # of consultations for diarrhea | # of total consultations for acute conditions | age (> / < 5) | HIS |
| 8.8 | Measles (trend of cases) per month | # of measles cases |  | age (> / < 5) | HIS |
| 8.9 | 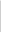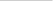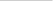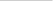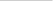Typhoid Fever (trend of cases) per month | # of Typhoid fever cases |  | age (> / < 5) | HIS |
| 8.10 | Meningitis (trend of cases) per month | # of Meningitis cases |  | age (> / < 5) | HIS |
| 8.11 | Polio/Flaccid Paralysis (trend of cases) per month | # of FP/Polio cases |  | age (> / < 5) | HIS |
| 8.12 | 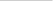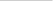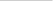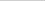Yellow fever (trend of cases) per month | # of Yellow fever cases |  | age (> / < 5) | HIS |
| 8.13 | Diabetes (trend of cases) per month | # of new diabetes cases |  |  | HIS |
| 8.14 | Diabetes (% morbidity) | # of consultations for new and old diabetes cases | # of consultations for chronic conditions at HF, all ages |  | HIS |
| 8.15 | Hypertension (trend of cases) per month | # of new hypertension cases, all ages |  |  | HIS |
| 8.16 | Hypertension (% morbidity) | # of consultations for new and old hypertension cases | # of consultations for chronic conditions at HF, all ages |  | HIS |
| 8.17 | HIV^[[10]](#footnote-10)^ - # of people tested per month | # of people tested for HIV per month |  | age (> / < 18), sex | HIS |
| 8.18 | HIV - # of positive tests per month | # of people who tested positive for HIV per month |  | age (> / < 18), sex | HIS |
| 8.19 | HIV - Trends in viral load levels (individual patient data) |  |  |  | individual level data |
| 8.20 | HIV - # of people enrolled in treatment program per month | # of people enrolled in HIV treatment program per month |  |  | HIS |
| 8.21 | TB - # of people enrolled in treatment program per month | # of patients enrolled in TB treatment program per month |  |  | HIS |
| 8.22 | TB - # of patients who completed treatment | # of patients who completed treatment in the month | # of patients who were expected to finish the treatment per month |  | HIS |
| 8.23 | Prevalence of Global acute malnutrition^[[11]](#footnote-11)^ among children 6 to 59 months | # of children with WHZ < -2 SD or MUAC <125mm (GAM), o BP oedema; | Estimated # of children under 5 in the area | sex | HIS |
| 8.24 | Prevalence of severe acute malnutrition among children 6 to 59 months | # of children with WHZ < -3 or MUAC<115 mm, or BP oedema (SAM) | Estimated # of children under 5 in the area | sex | HIS |
| 8.25 | Global Acute malnutrition (% morbidity) | # of children with WHZ < -2 SD or MUAC <125mm (GAM), o BP oedema; | # of U5 consultations | sex | HIS |
| 8.26 | Severe Acute malnutrition (% morbidity) | # of children with WHZ < -3 or MUAC<115 mm, or BP oedema (SAM) | # of U5 consultations | sex | HIS |
| **9. Mortality** | | | | | |
| 9.1 | Crude mortality: # of deaths recorded at the health facility (all age, all causes) | # of all-age, all-cause deaths that occur in a given population per unit of time or as captured in institutional records^[[12]](#footnote-12)^ | Population estimate | sex | HIS + population estimates |
| 9.2 | Under five mortality: # of deaths recorded at the health facility among children under the age of 5 (all causes) | # of under 5 all-cause deaths that occur in a given population per unit of time or as captured in institutional records^[[13]](#footnote-13)^ | Population estimate of children under 5 |  | HIS + population estimates |
| 9.3 | Maternal mortality - Female deaths from any cause related to or aggravated by pregnancy (up to 42 days after termination of pregnancy) | # of maternal deaths as captured in institutional records^[[14]](#footnote-14)^ | total # of deliveries in the institution |  | HIS |
| 9.4 | Still birth (baby born with no signs of life at or after 28 weeks' gestation) | # of stillbirths as captured in institutional records^[[15]](#footnote-15)^ |  |  | HIS |
| 9.5 | Neonatal (death occurring within 28 days from birth) | # of neonatal deaths as captured in institutional records^[[16]](#footnote-16)^ |  |  | HIS |
| 9.6 | Death due to Severe Acute Respiratory Infection | # of deaths from Severe Acute Respiratory Infections, all-age |  |  | HIS |

1. Contact settings in Jordan include family member, school, work, neighborhood. [↑](#footnote-ref-1)
2. Inclusion of this indicator to be confirmed once the isolation strategy in each site is clarified. [↑](#footnote-ref-2)
3. In Jordan, only data on ECMO available (no standard oxygen therapy via cannula) [↑](#footnote-ref-3)
4. Depending on data availability the definition will be confirmed in terms of scope (only emergency or all inpatient bed) and causes for occupation (all or COVID only). [↑](#footnote-ref-4)
5. Definition to be confirmed according to data availability in terms of included categories and frequency of reporting. [↑](#footnote-ref-5)
6. Which antibiotic will be used as tracer indicator will be decided based on data availability and definition used in UNHCR’s HIS [↑](#footnote-ref-6)
7. Whether only adult or pediatric treatment for malaria will be used will depend on data availability [↑](#footnote-ref-7)
8. Availability of data for Jordan camps still pending as data from UNICEF and not directly managed by UNHCR [↑](#footnote-ref-8)
9. Malaria not relevant in Jordan and therefore excluded [↑](#footnote-ref-9)
10. HIV excluded in Jordan. [↑](#footnote-ref-10)
11. Data on child malnutrition to be confirmed in Jordan as not routinely measured as last survey reported very low prevalence of SAM. [↑](#footnote-ref-11)
12. In Jordan, this includes deaths occurring outside of health facilities. [↑](#footnote-ref-12)
13. In Jordan, this includes deaths occurring outside of health facilities. [↑](#footnote-ref-13)
14. In Jordan, this includes deaths occurring outside of health facilities. [↑](#footnote-ref-14)
15. In Jordan, this includes deaths occurring outside of health facilities. [↑](#footnote-ref-15)
16. In Jordan, this includes deaths occurring outside of health facilities. [↑](#footnote-ref-16)
