# Supplementary material for: COVID-19 epidemiology and changes in health service utilization in Azraq and Zaatari refugee camps in Jordan: A retrospective cohort study
Source: PLoS Med. 2022 May 10;19(5):e1003993. doi: 10.1371/journal.pmed.1003993 (PMC9089859; doi:10.1371/journal.pmed.1003993)
Supplement: S3 Supporting Information — Table A: Interrupted times series results from alternative estimation models for RTIs, Azraq and Zaatari camps, Jordan. Fig A: Interrupted time series for diabetes consultations, Azraq and Zaatari camps, 2018 to 2021. Fig B: Interrupted time series of reproductive health indicators, Azraq and Zaatari camps, Jordan, January 1, 2018 to April 2, 2021: All family planning consultations (panels A and B); new family planning consultations (panels C and D). Table B: Proportion of COVID-19 cases by exposure type (all cases and by age groups), Azraq camp. Table C: Proportion of cases by setting of contact with other COVID-19 cases, by sex, Azraq camp. Table D: Proportion of COVID-19 cases by exposure type (all cases and by age groups), Zaatari camp. Table E: Proportion of cases by setting of contact with other COVID-19 cases, by sex, Zaatari camp. Table F: Average number of contacts per COVID-19 case, by sex and age groups, Azraq camp. Table G: Average number of contacts per COVID-19 case, by sex and age groups, Zaatari camp. Table H: Proportion of contacts followed by sex and age group of the case, Azraq camp. Table I: Proportion of contacts followed by sex and age group of the case, Zaatari camp. COVID-19, Coronavirus Disease 2019; RTI, respiratory tract infection. (DOCX) [file pmed.1003993.s003.docx]

**S3 Additional Results**

Table of Contents

[1 Respiratory tract infections – Sensitivity analysis 1](#_Toc101770233)

[2 Non-Communicable Diseases 2](#_Toc101770234)

[3 Reproductive health – Family planning indicators 3](#_Toc101770235)

[4 Exposure 4](#_Toc101770236)

[5 Contact tracing 5](#_Toc101770237)

# Respiratory tract infections – Sensitivity analysis

Table A: Interrupted Times Series results from alternative estimation models for Respiratory tract infections, Azraq and Zaatari camps, Jordan.

|  |  | **Azraq** | | **Zaatari** | |
| --- | --- | --- | --- | --- | --- |
|  |  | IRR immediate effect [95% CI] | IRR change in trend [95% CI] | IRR immediate effect [95% CI] | IRR change in trend [95% CI] |
| LRTI | gam | 1.159  [0.877 – 1.532] | 0.915  [0.885 – 0.945] | 1.597 [0.977 – 2.612] | 0.783  [0.740 – 0.829] |
|  | ARIMA | 0.986  [0.742 – 1.311] | 0.918  [0.884 – 0.954] | 1.423  [0.866 – 2.338] | 0.780  [0.729 – 0.835] |
|  | gls | 1.048  [0.585 – 1.601] | 0.919  [0.873 – 0.967] | 1.423  [0.721 – 2.845] | 0.787  [0.727 – 0.852] |
| URTI | gam | 0.693  [0.561 – 0.855] | 0.971  [0.947 – 0.995] | 0.604  [0.446 – 0.818] | 0.972  [0.937 – 1.007] |
|  | ARIMA | 0.626  [0.488 – 0.802] | 0.983  [0.950 – 1.017] | 0.657  [0.477 – 0.905] | 0.982  [0.942 – 1.025] |
|  | gls | 0.685  [0.524 – 0.894] | 0.973  [0.942 – 1.004] | 0.553  [0.331 – 0.923] | 0.988  [0.925 – 1.054] |
| ILI | gam | 0.915  [0.588 – 1.425] | 0.874  [0.832 – 0.911] | 0.668  [0.480 – 0.929] | 0.873  [0.839 – 0.908] |
|  | gam (gaussian) | 0.764  [0.354 – 1.649] | 0.891  [0.801 – 0.992] | 0.673  [0.383 – 1.183] | 0.892  [0.808 – 0.984] |
|  | ARIMA | 0.867  [0.588 – 1.277] | 0.880  [0.835 – 0.928] | 0.498  [0.335 – 0.740] | 0.902  [0.858 – 0.948] |
|  | gls | 0.821  [0.483 – 1.393] | 0.881  [0.826 – 0.939] | 0.594  [0.335 – 1.051] | 0.891  [0.827 – 0.960] |
| All RTI | gam | 0.749  [0.596 – 0.940] | 0.949  [0.924 – 0.975] | 0.625  [0.641 – 0.849] | 0.936  [0.904 – 0.970] |
|  | ARIMA | 0.692  [0.532 – 0.90] | 0.958  [0.924 – 0.993] | 0.540  [0.369 – 0.778] | 0.951  [0.906 – 0.998] |
|  | gls | 0.733  [0.545 – 0.985] | 0.952  [0.919 – 0.986] | 0.572  [0.346 – 0.946] | 0.950  [0.894 – 1.009] |

# Non-Communicable Diseases


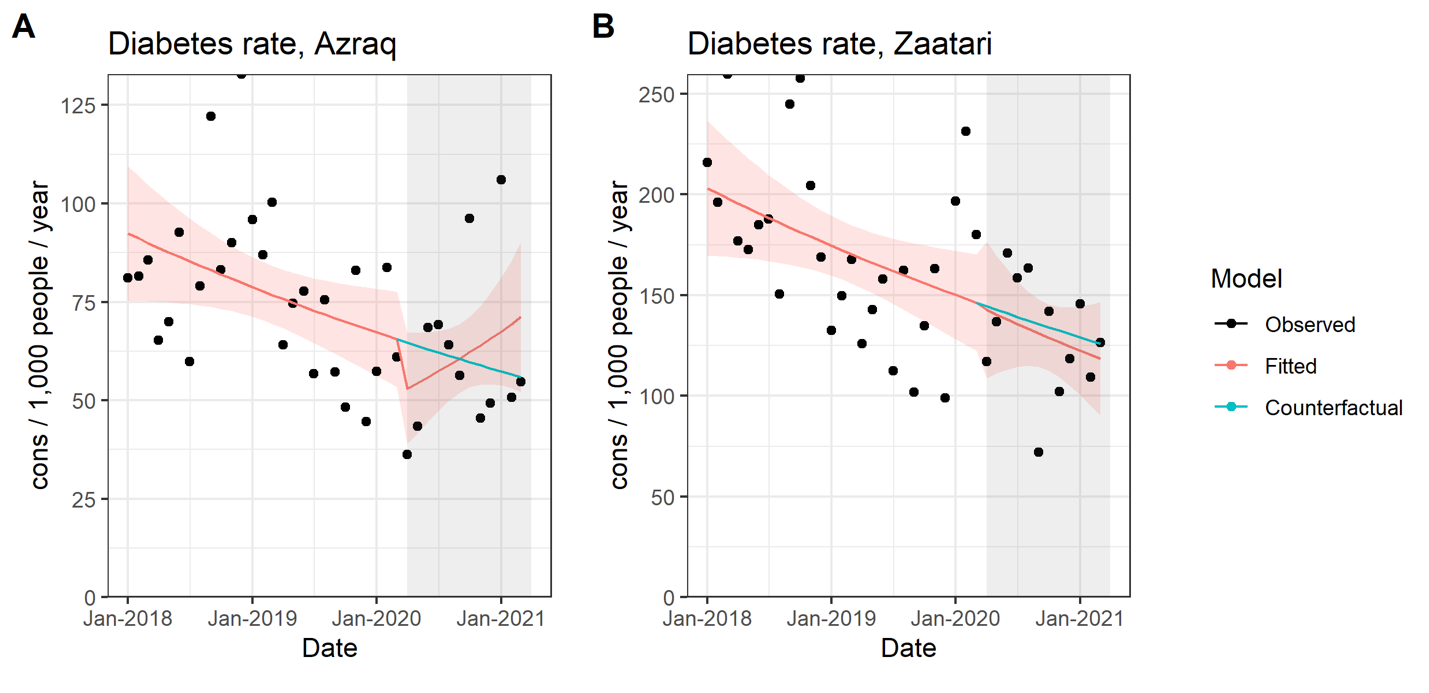


Figure A: Interrupted time series for diabetes consultations, Azraq and Zaatari camps, 2018 to 2021.

# Reproductive health – Family planning indicators


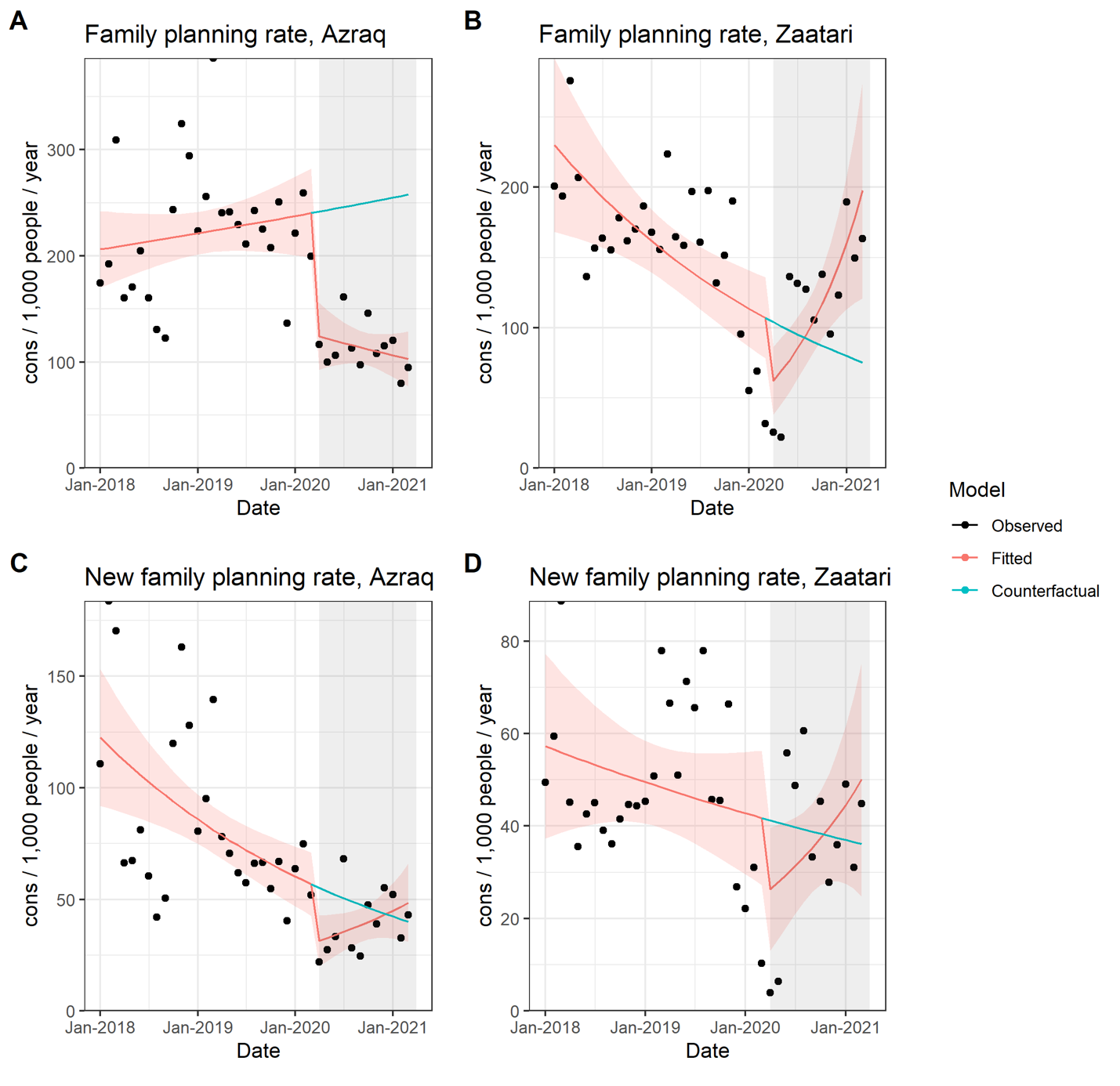


Figure B: Interrupted time series of reproductive health indicators, Azraq and Zaatari camps, Jordan, January 1, 2018- April 2, 2021: All family planning consultations (panels A and B); new family planning consultations (panels C and D).

# Exposure

Table B: Proportion of COVID-19 cases by exposure type (all cases and by age groups), Azraq camp

|  | | | **Overall** | | **Female** | | | | **Male** | | | **p** | |
| --- | --- | --- | --- | --- | --- | --- | --- | --- | --- | --- | --- | --- | --- |
| n | | | 901 | | 518 | | | | 383 | | |  | |
| HCW (%) | | | 15 (1.7) | | 6 (1.2) | | | | 9 (2.3) | | | 0.263 | |
| Traveled in past 14 days* (%) | | | 93 (10.3) | | 54 (10.4) | | | | 39 (10.2) | | | 0.994 | |
| Visited Health Facility (%) | | | 69 (7.7) | | 38 (7.3) | | | | 31 (8.1) | | | 0.767 | |
| Contact (%) | | | 91(10.1) | | 57 (11.0) | | | | 34 (8.9) | | | 0.350 | |
|  | **Overall** | **0-4** | **5-11** | **12-17** | | **18-29** | **30-39** | **40-49** | | **50-59** | **60+** | | **p** |
| n | 901 | 69 | 150 | 95 | | 167 | 208 | 119 | | 44 | 49 | |  |
| Traveled* | 93 (10.3) | 9 (13.0) | 8 (5.3) | 12 (12.6) | | 29 (17.4) | 9 (4.3) | 18 (15.1) | | 3 (6.8) | 5 (10.2) | | 0.001 |
| Visited HF | 69 (7.7) | 5 (7.2) | 6 (4.0) | 1 (1.1) | | 23 (13.8) | 19 (9.1) | 7 (5.9) | | 2 (4.5) | 6 (12.2) | | 0.004 |
| Contact | 91 (10.1) | 7 (10.1) | 35 (23.3) | 17 (17.9) | | 7 (4.2) | 11 (5.3) | 7 (5.9) | | 4 (9.1) | 3 (6.1) | | <0.001 |

Table C: Proportion of cases by setting of contact with other COVID-19 cases, by sex, Azraq camp

|  | **Overall** | **Female** | **Male** | **p** |
| --- | --- | --- | --- | --- |
| n | 91 | 57 | 34 |  |
| Contact Setting (%) |  |  |  | NaN |
| Family | 34 (38.2) | 18 (32.1) | 16 (48.5) |  |
| Work | 0 (0.0) | 0 (0.0) | 0 (0.0) |  |
| School | 0 (0.0) | 0 (0.0) | 0 (0.0) |  |
| Neighbor | 55 (61.8) | 38 (67.9) | 17 (51.5) |  |
| Other | 0 (0.0) | 0 (0.0) | 0 (0.0) |  |
| Unknown | 0 (0.0) | 0 (0.0) | 0 (0.0) |  |

Table D: Proportion of COVID-19 cases by exposure type (all cases and by age groups), Zaatari camp

|  | | | | **Overall** | | | **Female** | | | **Male** | | | **p** | | | |  |  |
| --- | --- | --- | --- | --- | --- | --- | --- | --- | --- | --- | --- | --- | --- | --- | --- | --- | --- | --- |
| n | | | | 1715 | | | 949 | | | 766 | |  | | | |  |  |  |
| HCW (%) | | | | 31 (1.8) | | | 13 (1.4) | | | 18 (2.3) | | 0.183 | | | | | |  |
| Traveled in past 14 days* (%) | | | | 62 (3.6) | | | 39 (4.1) | | | 23 (3.0) | | 0.275 | | | | | |  |
| Visited Health Facility (%) | | | | 205 (12.0) | | | 127 (13.4) | | | 78 (10.2) | | 0.050 | | | | | |  |
| Contact (%) | | | | 1034 (60.3) | | | 575 (60.6) | | | 459 59.9) | | 0.817 | | | | | |  |
|  | **Overall** | **0-4** | **5-11** | | **12-17** | **18-29** | | **30-39** | **40-49** | | **50-59** | | | **60+** | **p-value** | | | |
| n | 1715 | 96 | 313 | | 235 | 360 | | 278 | 217 | | 130 | | | 86 |  | | | |
| Traveled | 62 (3.6) | 0 (0.0) | 0 (0.0) | | 3 (1.3) | 16 (4.4) | | 16 (5.8) | 12 (5.5) | | 6 (4.6) | | | 9 (10.5) | <0.001 | | | |
| Visited HF | 205 (12.0) | 1 (1.0) | 13 (4.2) | | 10 (4.3) | 45 12.5) | | 53 19.1) | 35 (16.1) | | 19 (14.6) | | | 29 (33.7) | <0.001 | | | |
| Contact | 1034 (60.3) | 93 (96.9) | 272 (86.9) | | 197 (83.8) | 180 (50.0) | | 117 (42.1) | 85 (39.2) | | 66 (50.8) | | | 24 (27.9) | <0.001 | | | |

Table E: Proportion of cases by setting of contact with other COVID-19 cases, by sex, Zaatari camp

|  | **Overall** | **Female** | **Male** | **p** |
| --- | --- | --- | --- | --- |
| n | 1034 | 575 | 459 |  |
| Contact Setting (%) |  |  |  | <0.001 |
| Family | 877 (84.8) | 501 (87.1) | 376 (81.9) |  |
| Work | 72 (7.0) | 15 (2.6) | 57 (12.4) |  |
| School | 35 (3.4) | 31 (5.4) | 4 (0.9) |  |
| Neighbor | 3 (0.3) | 2 (0.3) | 1 (0.2) |  |
| Other | 4 (0.4) | 1 (0.2) | 3 (0.7) |  |
| Unknown | 43 (4.2) | 25 (4.3) | 18 (3.9) |  |

# Contact tracing

Table F: Average number of contacts per COVID-19 case, by sex and age groups, Azraq camp

|  | **Total** | **Female** | **Male** | **0-4** | **5-11** | **12-17** | **18-29** | **30-39** | **40-49** | **50-59** | **60+** |
| --- | --- | --- | --- | --- | --- | --- | --- | --- | --- | --- | --- |
| n | 900 | 518 | 382 | 69 | 150 | 95 | 166 | 208 | 119 | 44 | 49 |
| Mean | 4.9 | 4.7 | 5.3 | 4.6 | 5.2 | 5.3 | 4.7 | 5.3 | 5.4 | 3.2 | 3.0 |
| Median | 5 | 5 | 5 | 4.5 | 6.0 | 4.0 | 5.0 | 5.0 | 5.0 | 3.0 | 5.0 |
| Q1 | 3 | 3 | 3 | 2.2 | 3.8 | 3.5 | 3.0 | 3.2 | 4.0 | 2.2 | 0.0 |
| Q3 | 6 | 6 | 7 | 6.8 | 6.0 | 6.0 | 5.0 | 7.0 | 7.0 | 3.8 | 5.0 |
| p-value |  | 0.19 | | 0.24 | | | | | | | |

Table G: Average number of contacts per COVID-19 case, by sex and age groups, Zaatari camp

|  | **Total** | **Female** | **Male** | **0-4** | **5-11** | **12-17** | **18-29** | **30-39** | **40-49** | **50-59** | **60+** |
| --- | --- | --- | --- | --- | --- | --- | --- | --- | --- | --- | --- |
| n | 1715 | 949 | 766 | 96 | 313 | 235 | 360 | 278 | 217 | 130 | 86 |
| Mean | 5.7 | 5.7 | 5.6 | 5.6 | 6.6 | 6.8 | 5.0 | 5.6 | 5.8 | 4.2 | 4.0 |
| Median | 5 | 5 | 5 | 5 | 6 | 7 | 4 | 5 | 6 | 4 | 2 |
| Q1 | 4 | 4 | 4 | 4 | 5 | 5 | 3 | 4 | 4 | 2 | 1 |
| Q3 | 7 | 7 | 7 | 7 | 8 | 8 | 6 | 7 | 7 | 6 | 5 |
| p-value |  | 0.37 | | 0 | | | | | | | |

Table H: Proportion of contacts followed by sex and age group of the case, Azraq camp

|  | **Total** | **F** | **M** | **0-4** | **5-11** | **12-17** | **18-29** | **30-39** | **40-49** | **50-59** | **60+** |
| --- | --- | --- | --- | --- | --- | --- | --- | --- | --- | --- | --- |
| Followed | 456 | 245 | 211 | 57 | 129 | 56 | 72 | 70 | 52 | 15 | 5 |
| Contacts | 681 | 405 | 276 | 82 | 167 | 80 | 98 | 117 | 103 | 19 | 15 |
| Proportion | 67.0 | 60.5 | 76.4 | 69.5 | 77.2 | 70.0 | 73.5% | 59.8% | 50.5% | 78.9% | 33.3% |

Table I: Proportion of contacts followed by sex and age group of the case, Zaatari camp

|  | **Total** | **F** | **M** | **0-4** | **5-11** | **12-17** | **18-29** | **30-39** | **40-49** | **50-59** | **60+** |
| --- | --- | --- | --- | --- | --- | --- | --- | --- | --- | --- | --- |
| Followed | 9533 | 5330 | 4203 | 520 | 2041 | 1586 | 1776 | 1522 | 1235 | 532 | 321 |
| Contacts | 9567 | 5356 | 4211 | 520 | 2048 | 1593 | 1783 | 1528 | 1239 | 535 | 321 |
| Proportion | 99.6 | 99.5 | 99.8 | 100.0 | 99.7 | 99.6 | 99.6 | 99.6 | 99.7 | 99.4 | 100.0 |
