# Supplementary material for: COVID-19 epidemiology and changes in health service utilization in Azraq and Zaatari refugee camps in Jordan: A retrospective cohort study
Source: PLoS Med. 2022 May 10;19(5):e1003993. doi: 10.1371/journal.pmed.1003993 (PMC9089859; doi:10.1371/journal.pmed.1003993)
Supplement: S4 Supporting Information — NPI, nonpharmaceutical intervention. (DOCX) [file pmed.1003993.s004.docx]

**S4. Non-pharmaceutical Interventions introduced in refugee camps in Jordan**

Table A: Timeline of major events in Jordan and Public Health and Social Measures implemented in Azraq and Zaatari camps, March 2020 to March 2021.

| **Date** | **Public Health and Social Measures** | **Holiday** |
| --- | --- | --- |
| **2020** |  |  |
| 21-Mar | Start stay at home order in all regions and cities of the kingdom and all shops closed |  |
| 24-Mar | [Zaatari] shops open 10 to 18; schools closed |  |
| 25-Mar | [Zaatari] all movements in camps prohibited; gathering of any size not permitted; physical distancing |  |
| 31-Mar | Any type of distribution in camps is suspended; UNICEF will distribute soap |  |
| 1-Apr | Shops will be closed Fridays (from April 10th, Fridays and Saturdays) |  |
| 5-Apr | Every vehicle entering the camp is sterilized |  |
| 6-Apr | UNHCR & NCR will start distributing cash to cover the needs for cooking, sanitary napkins, baby diapers |  |
| 8-Apr | Hand sanitization of all PoC and staff entering the site; distribution from two different locations to reduce crowding; physical distancing while queuing; continuous cleaning and disinfection of surface. |  |
| 11-Apr | Following GoJ directive, 150 random tests will be undertaken in Zaatari camp for different age groups. |  |
| 23-Apr | Total curfew on Friday April 24 for 24 hours; stay at home order + all shops closed | Ramadan April 23 to May 23 2020 |
| 27-Apr | Camps still closed |  |
| 30-Apr | Vaccination activities in camps have been resumed |  |
| 11-May | Traders, shop owners, gas sellers and other service providers are expected to wear face masks/face coverings and gloves especially while interacting with customers; 10GB internet bundles will be recharged for all families who have children in schools |  |
| 12-May | Ramadan gatherings are not permitted |  |
| 14-May | Appointments in the clinics are given based on the priority and according to appointments system, in align with MoH instruction not to gather high numbers in the clinics. Therefore, few numbers will be treated inside the clinics within working hours. |  |
| 19-May | The first day of Eid will be a full lockdown. The movement on the second day of Eid will be allowed only on foot. |  |
| 21-May | Market, supermarkets closed Friday to Monday 22 to 24 |  |
| 22-May | Curfew will be imposed from 22 to morning 25 |  |
| 23-26  May |  | Eid-al-Fitr holiday |
| 4-Jun | Curfew were applied from June 5 to June 6; movement within camp allowed from 11am to 2pm for prayer in two designated areas (Sport House and the Taekwondo academy). All mosques in the camps will be closed; children <14 forbidden to prayers; elderly or those suffering from any diseases should stay home; everyone should wear a mask and gloves; own prayer rug; physical distancing; no shaking hands; police officers will be present. |  |
| 11-Jun | Curfew is applied on daily basis from midnight to 6 am; otherwise, refugees are allowed to move within the camp; prayer conditions as per June 4 |  |
| 21-Jul | UNHCR starts remote registration services through phone calls. |  |
| 27-Jul | Restoring the system of work permits for refugee residing in the camp; leave permits will be granted to refugees residing in the camp for the special cases that require to leave; refugees currently outside the camps can return to the camp. Temperature will be measured at the gate |  |
| 30 – Jul  to 3 -Aug |  | Eid-al-Adha holidays |
| 9-Aug | Curfew reduced to 2am to 6 am; exit and entry of refugees who had a permit; Jordan authority encourages downloading the AMAN app |  |
| 11-Aug | From August 15, every person must adhere to social distance and wearing a mask when entering public places or places where services are provided; health care providers are obliged to wear a mask and gloves, visitors must wear masks too; sudden visits to public places will be done to ensure compliance. |  |
| 13-Aug | From august 15, curfew from 12am to 6 am + civilian must wear a mask when in public |  |
| 20 -Aug |  | Islamic New Year |
| 24-Aug | Curfew from 10pm to 6am |  |
| 27-Aug | [Amman + Zarqa governorate]: total curfew August 28 for 24 hours, stay home + all shops closed; from august 29, curfew from 11pm to 6am |  |
| 28-Aug | Caritas clinics welcome the public only by appointment |  |
| 30-Aug | Curfew 10pm to 6am only. School reopening: 50% capacity with shifts according to grade; masks distributed by UNICEF, classroom sterilized |  |
| 9-Sep | [Azraq]: School still closed; work and leave permits suspended; physical distance + wearing a mask; children in the entire kingdom are exempted from the donation fee and cost of books. |  |
| 10-Sep | [Zaatari] a case of COVID has been found in the camp; contact tracing teams of MoH will be in the camp; random tests; only essential movements; |  |
| 12-Sep | Defense order 8: all individuals must immediately notify competent authorities if they are infected and if they have come into close contact with a case; adhere to home quarantine and lockdown; obligation on all hospitals, medical centers and medical laboratories to disclose the presence of covid cases. |  |
| 12-Sep | Defense Order 11: obligation to wear mask and adhere to physical distancing in private and public spaces where services are provided directly to people. |  |
| 12-Sep | [Zaatari]: School will be closed for a week starting sept 13; remote learning. All community centers also suspended; work and leave permits suspended; mosques closed for a week; essential movements; refugees who are outside can get back latest by Sept 14. |  |
| 12-Sep | [Azraq]: School suspended for a week; remote learning; community centers suspended for a week; work and leave permits are suspended; physical distancing enforced; essential movements only; sending rumors has legal consequences |  |
| 14-Sep | [Zaatari]: Sterilization campaign conducted by UNHCR/UNICEF night of Sept 14; curfew from midnight to 6 AM only; physical distancing, public hygiene, commitment to wearing masks, essential movements only, legal consequences for sending rumors. |  |
| 15-Sep | As of Sept 17, and for two weeks, all mosques and churches shutdown and prayer suspended; public and private schools switched to distant learning, electricity hours changed to accommodate online education hours from 9 AM – 11 PM [Zaatari] and 9 AM – 2 AM [Azraq]; restaurants/ cafes/ shutdown with only pick-up or delivery services possible; popular and central markets shut down; large event gatherings strictly prohibited; curfew 1 AM to 6 AM; ‘lockdown Fridays’ remain lifted unless announced otherwise |  |
| 1-Oct | [Zaatari]: As of Oct 1, and for two weeks, in-school classes suspended from fourth grade to eleventh grade. For Tawjihi students, school attendance ongoing, and students of KG2s, first to third grade, choice is up to parents. Community centers suspended, work/leave permits suspended, large gatherings still prohibited, mosques opened for prayers with respect to all precaution measures and defense order by gov; curfew from midnight to 6 AM, and previous instructions/ rules regarding distance, hygiene, and masks remain; school have been disinfected previously and desks have been positioned in a way that guarantees social distancing. |  |
| 1-Oct | [Azraq]: Mosques in the open village 2, 3, and 6 will be closed but remained open for Friday prayers only and remained closed for rest of prayers. Village 5 mosques closed for all prayers until further notice; work continued inside markets in all villages for shops that provide direct sales services only; work in community centers suspended. |  |
| 6-Oct | [Zaatari]: cases of infection found among refugees living in the camp: the refugees will be transferred along with their families to the transit area; expanded movement of MOH tracing teams inside camp and refugees will be randomly tested; only essential movements. |  |
| 7-Oct &  14 Oct &  21-Oct | [Zaatari]: Starting midnight Oct 8, full lockdown during the weekends, school/ kindergartens suspended and transferred to distance education, attendance of teachers and administrative bodies in public schools by rotation system, and public/private university attendance remote; bakeries/bread distribution centers allowed to open only on Saturdays 8 AM – 2 PM; a max of 3 kg of bread will be sold to each family; all refugees under home quarantine must not leave their homes; free transportation WFP used to provide to supermarket has been stopped until further notice entry and exit through the berm strictly monitored and prohibited for all ages- SRAD has increased surveillance at the berm |  |
| 7-14-21-28 Oct | [Azraq]: on Fridays, all markets and bread selling centers will be completely closed, but will be continued on Saturdays, 10/10 and 17/10 from 8 AM to 2 PM. Markets in all villages in camp will be closed during lockdown period and work remains suspended in community centers; hours of electricity supply changed from 9 AM to 11 PM; previous, standard measures/ instructions remain; UNICEF working to provide parents of students in the camp’s schools internet packages to facilitate educational platforms; UNICEF asks that health facilities allocate one bathroom for infected and one for non-infected |  |
| 21-Oct | [Zaatari]: as a result of the infection of one of the staff working in the Sharia court inside the camp, work of Sharia court suspended until further notice. |  |
| 22-Oct | [Zaatari + Azraq]: movement of people from 12:15 until 1:15 PM on Friday for Friday prayers allowed under the conditions: period is used for worship only, elderly and children under 15 advised not to go, general safety measures must be taken, the prayer will not exceed 10 minutes, after 1:15 the curfew returns, and that no one is allowed to walk inside the camp during this hour |  |
| 28-Oct | [Azraq]: on occasion of Prophet’s birthday, clinics will be closed on Thursday; previous instructions/measures remain regarding markets being closed, suspension of work permits/licenses, and mask wearing |  |
| 28-Oct  to dec 2020 | [Zaatari]: Full lockdown applied to all camp residents on weekends until the end of 2020; daily curfew 10 PM to 6 AM; movement inside camp prohibited during Fridays, markets and non-official shops inside camp will be closed throughout the days of the lockdown; movement for prayer time continues but time window changed to new wintertime 11:15 AM – 12:15 PM; previous instructions/ measures remain |  |
| 29- Oct |  | Prophet's birthday |
| 2-Nov | [Zaatari]: Policemen in all governorates will begin issuing fines against individuals violating the Defense Law in facilities, public places, markets, etc.; night curfew modified to be from 9 PM to 6 AM; all refugees under home quarantine must not leave their homes under any circumstances; entry/exit through the berm strictly monitored and prohibited for all ages |  |
| 9-Nov | [Zaatari]: Full lockdown applied to all camp residents starting 9 PM on Nov 10 until 6 AM of Nov 15; movement inside camp prohibited, but it will still be allowed to approach the two supermarkets and bread distribution centers on Nov 12+14, from 8 AM until 2 PM; non-official shops inside camp will be closed throughout the lockdown |  |
| 10-Nov |  | Elections |
| 11-Nov | [Zaatari]: Notice for death of a refugee with “underlined health conditions”; re-enforcing all instructions/measures |  |
| 16-Nov | [Zaatari]: Defense order notice noting work for Sharia court suspended from Nov 15 until Nov 25, and that court will not receive refugees until that date; |  |
| 25-Nov to  End of Dec | [Azraq]: on Fridays, all markets and bread selling centers will be completely closed, noting they will continue on Saturdays; Friday prayer will be allowed from 11:15-12:15 PM with aforementioned conditions; markets closed during lockdown period, suspension of work in community centers and suspension of permits/ licenses, and mask wearing |  |
| 8-Dec | [Zaatari]: Notice that UNHCR is providing small rooms to high-risk population members with severe health conditions |  |
| 15-Dec | [Zaatari]: WFP, starting Dec 16, will change the quality of food provided to the refugees residing in isolation areas by distributing new food materials- the food parcel will be distributed to individuals in the isolation area |  |
| 25-Dec |  | Christmas |
| **2021** |  |  |
| 1-Jan |  | New Year |
| 1-Feb | [Zaatari]: Defense order 25: workers who fail to observe COVID-19 safety measures instituted by gov will be fined an amount ranging between JD20 and JDD50; if private sector and/or its employees violate the new clause, fines ranging between JD500 and JD1,000 |  |
| 6-Feb | [Zaatari]: Notice of instructions for school children attendance by grade |  |
| 9-Feb | [Zaatari]: Leave permit office resumes work; the following health measures applied to those who left the camp upon their return: increase the number of PCR at the entrance of the camp, and thermal inspection of those returning from the leaves if symptoms of infection developed; Syrian refugees leaving the camp should commit the safety measures and avoid gathering during their leave; Syrian refugees encouraged to apply on MOH platform to receive COVID-19 vaccine |  |
| 11-Feb | [Zaatari]: Refugees are eligible to register for COVID-19 vaccine through MOH platform |  |
| 16-Feb | [Azraq]: Attendance in schools suspended Feb 17 and 18, distance learning. |  |
| 24-Feb &  3 Mar | [Azraq]: on Fridays, all markets and bread selling centers will be completely closed, continue on Saturdays; re-emphasis on Friday prayer rules; isolated neighborhoods and all individuals under home isolation are excluded from the prayer rules/decision |  |
| 11-Mar | [Azraq]: Friday prayers have been suspended till further notice, partial ban hours have been extended to start from 7 PM, and closing of shops in market starting at 6 PM |  |
|  |  |  |
