# Supplementary material for: COVID-19 epidemiology and changes in health service utilization in Azraq and Zaatari refugee camps in Jordan: A retrospective cohort study
Source: PLoS Med. 2022 May 10;19(5):e1003993. doi: 10.1371/journal.pmed.1003993 (PMC9089859; doi:10.1371/journal.pmed.1003993)
Supplement: Attachment — Submitted filename: PLOS_Article_Refugees_210222.docx [file pmed.1003993.s005.docx]

**Reviewer Recommendation and Comments**

**Manuscript Number PMEDICINE-D-22-00341R1**

**COVID-19 pandemic in Azraq and Zaatari refugee camps in Jordan: are refugees in camps at reduced risk?**

Revision Number 1

**Michel Thieren,** WHO Representative to Israel; Special Adviser to the WHO Regional Director for Europe on COVID-19

-------------------------------------------

- The article bares very little comments. It is comprehensive, quite unique in its focus, is well documented and referenced, with a rich set of conclusions which interestingly are not necessarily the intuitive ones readers would expect.
- Authors should therefore be praised for this detailed work in collecting and processing this innovative set of evidence on refugee camps in times of COVID-19
- The article may however provide a misperception about refugees in closed camps setting as if such camps conditions constitute in themselves “counter-measures” for pandemic spread. The various biases and limitations in the interpretation of the data set are very well explained, but a reminder that the situation in these camps should not be seen as pandemic mitigation factors and generalized as such. It is obvious that this would not be the intention of the authors, but it could be a side effect of the way these conclusions are presented – without cautioning statement. A sentence or two should therefore warn against any arguments equating refugee protection condition to refugee health. It is Jordan’s and UNHCR merit to ensure universal access to adequate health services to refugees in these camps, but these are still ‘camps’ with living conditions marked by severely restricted liberties to be *no* justification for protection against COVID-19.
- Although vaccines were not available at the time of the study, the conclusions of COVID-19 being a milder problem (than expected) in refugee camp settings may be misused politically to delay access to vaccines for refugees nowadays. This may be off-subject here, but a sentence should highlight that – given the parallel in this data set with what we know of COVID-19 -vaccination is likely to hold the same benefit in refugee camps than anywhere else, and that refugee camps should remain a top priority for any national COVID-19 vaccination roll-out.
- The impact of NPIs in camp setting, which can be implemented more easily than in open societies, is well mentioned, but would deserve more elaboration: it could be good to have (if possible) them – if not quantified (stringency index overtime) – at least better qualified, so that we can have a better understanding of their greater impact in closed camps than outside them.
- Finally, the favorable course of the pandemic in these two camps compared to the outside communities in Jordan cannot be replicated to migrant camp settings such in Grece. This is evidently another subject, but in the commentary section, a couple of sentences contrasting the two situations could be added. Reference to: S Hargreaves, E Kondilis, D Papamichail, S McCann, M Orcutt, E Carruthers, A Veizis, The impact of COVID-19 on migrants in Greece: a retrospective analysis of national data, European Journal of Public Health, Volume 31, Issue Supplement_3, October 2021, ckab164.589, <https://doi.org/10.1093/eurpub/ckab164.589> . Again, the intention is to avoid overinterpreting these conclusions to setting that may overlap but which bare very different assumptions in terms f protection, level of care, and state oversight.
- To mention a typo in the last sentence: “ there are no available data are available to analyze its effects ”
- **In conclusion:** a great article with important data and new evidence presented, deserving immediate publication without revisions, apart from cautioning against possible misuse or over/misinterpretation of some of the conclusions at political level.
