# Supplementary material for: COVID-19 epidemiology and changes in health service utilization in Azraq and Zaatari refugee camps in Jordan: A retrospective cohort study
Source: PLoS Med. 2022 May 10;19(5):e1003993. doi: 10.1371/journal.pmed.1003993 (PMC9089859; doi:10.1371/journal.pmed.1003993)
Supplement: Attachment — Submitted filename: Authors response to reviewers comments_031522.docx [file pmed.1003993.s006.docx]

**Comments to the article: COVID-19 pandemic in Azraq and Zaatari refugee camps in Jordan: are refugees in camps at reduced risk?"**

**(PMEDICINE-D-22-00341R1) for consideration at PLOS Medicine.**

Baltimore, March 15, 2022

Dear Editors and reviewers,

Thank you for your reviews and useful comments.

Please find below the point by point responses from the authors.

We remain at your disposal for additional clarifications if needed.

Best wishes,

Paul Spiegel

**Requests from the editors:**

**1) Please revise your title according to PLOS Medicine's style. Your title must be nondeclarative and not a question. It should begin with main concept if possible. Please place the study design in the subtitle (i.e., after a colon), e.g., a retrospective cohort study. Include setting in the title. For example, “COVID-19 epidemiology and health service utilization among refugees in Azraq and Zaatari refugee camps in Jordan: a retrospective cohort study”.**

Authors’ reply: Noted, we suggest following revised title:

COVID-19 epidemiology and changes in health service utilization in Azraq and Zaatari refugee camps in Jordan: a retrospective cohort study

**2) Is there a chance you can obtain more recent data from this setting especially after vaccine roll out?**

Authors’ reply: The study focused on the first year of the pandemic (i.e., between March 2020 and March 2021). By March 2021, vaccination had just started in Jordan and in the camps, and it would be difficult to include vaccination in the analysis as it did not cover the entire period. Expanding the period covered by the study to include vaccination would require extensive work to obtain the data as vaccines were administered by the Government of Jordan. While this is an important question that should be addressed in the future it would not be feasible in this short timeline.

**3) We note that you conducted research or obtained samples in a foreign country. Did you receive local IRB or ethical approval? Please check the relevant national regulations and laws applying to foreign researchers and state whether you obtained the required permits and approvals. Please address this in your ethics statement in both the manuscript and submission information.**

Authors’ reply: IRB approval was obtained from JHSPH which determined the study to be Non-Human Subject Research. No additional in-country authorization was required as all data are anonymized (COVID-19) and aggregated (routine health service).

**4) The Data Availability Statement (DAS) requires revision. For each data source used in your study, if the data are not freely available, please describe briefly the ethical, legal, or contractual restriction that prevents you from sharing it. Please also include an appropriate contact (web or email address) for inquiries (this cannot be a study author).**

Authors’ reply:

Because of ethical and legal considerations related to the vulnerability of the refugee populations, only de-identified individual-level data (COVID-19) and aggregate data (routine health services) may be made available upon request following publication and approval of a concept note summarizing the analyses to be conducted.

**5)      Abstract:**

a) **Please ensure that all numbers presented in the abstract are present and identical to numbers presented in the main manuscript text.**

Authors’ reply: Noted and done.

b) **Please include the population size**

Authors’ reply: Noted, camps population has been added.

c) **Please include the actual amounts and/or absolute risk(s) of relevant outcomes, not just coefficients.**

Authors’ reply: Abstract revised.

d) **Please quantify the main results (please present both 95% CIs and p values).**

Authors’ reply: Abstract revised.

e) **In the last sentence of the Abstract Methods and Findings section, please describe the main limitation(s) of the study's methodology.**

Authors’ reply: Abstract revised.

f) **Please address the study implications without overreaching what can be concluded from the data.**

Authors’ reply: Abstract revised.

**6) At this stage, we ask that you include a short, non-technical Author Summary of your research to make findings accessible to a wide audience that includes both scientists and non-scientists. The Author Summary should immediately follow the Abstract in your revised manuscript. This text is subject to editorial change and should be distinct from the scientific abstract.** Please see our author guidelines for more information:

<https://nam02.safelinks.protection.outlook.com/?url=https%3A%2F%2Fjournals.plos.org%2Fplosmedicine%2Fs%2Frevising-your-manuscript%23loc-author-summary&;data=04%7C01%7Cpbspiegel%40jhu.edu%7Cc11a229c41f94ee4cf3008d9fd541086%7C9fa4f438b1e6473b803f86f8aedf0dec%7C0%7C0%7C637819359772374136%7CUnknown%7CTWFpbGZsb3d8eyJWIjoiMC4wLjAwMDAiLCJQIjoiV2luMzIiLCJBTiI6Ik1haWwiLCJXVCI6Mn0%3D%7C3000&sdata=uBl539SGysiHTOad4zAwU%2BXcRyTgJS0d48HNbQD%2BeDQ%3D&reserved=0>

Authors’ reply: Added after the abstract (page 3).

**7) Please avoid assertions of primacy (“This is the first study to ..."). Instead, state “to our knowledge” or similar.**

Authors’ reply: Noted.

**8) Please remove the last sentence of your introduction (“We adjust our analyses …”) and move it the Methods section**

Authors’ reply: We do not see the indicated sentence in the introduction. No changes made.

**9) Did your study have a prospective protocol or analysis plan? Please state this (either way) early in the Methods section.**

a) **If a prospective analysis plan (from your funding proposal, IRB or other ethics committee submission, study protocol, or other planning document written before analyzing the data) was used in designing the study, please include the relevant prospectively written document with your revised manuscript as a Supporting Information file to be published alongside your study, and cite it in the Methods section. A legend for this file should be included at the end of your manuscript.**
b) **If no such document exists, please make sure that the Methods section transparently describes when analyses were planned, and when/why any data-driven changes to analyses took place.**
c) **In either case, changes in the analysis-- including those made in response to peer review comments-- should be identified as such in the Methods section of the paper, with rationale.**

Authors’ reply: We drafted an analysis plan during the study design phase. We have edited the methods section (page 5) and made reference to it. The analysis plan has been added into the supporting information.

**10) Please ensure that the study is reported according to the STROBE guideline, and include the completed STROBE checklist as Supporting Information.**

**Please add the following statement, or similar, to the Methods: "This study is reported as per the Strengthening the Reporting of Observational Studies in Epidemiology (STROBE) guideline (S1 Checklist)."**

**The STROBE guideline can be found here:**

<https://nam02.safelinks.protection.outlook.com/?url=http%3A%2F%2Fwww.equator-network.org%2Freporting-guidelines%2Fstrobe%2F&;data=04%7C01%7Cpbspiegel%40jhu.edu%7Cc11a229c41f94ee4cf3008d9fd541086%7C9fa4f438b1e6473b803f86f8aedf0dec%7C0%7C0%7C637819359772374136%7CUnknown%7CTWFpbGZsb3d8eyJWIjoiMC4wLjAwMDAiLCJQIjoiV2luMzIiLCJBTiI6Ik1haWwiLCJXVCI6Mn0%3D%7C3000&sdata=wrWLY0291zK0XYB%2Fa5QHlh7X77QzFpnzaCyza5q%2BRXg%3D&reserved=0>

**a)      When completing the checklist, please use section and paragraph numbers, rather than page numbers.**

Authors’ reply: Noted. Method section revised (page 5) and checklist added to the supporting information.

**11) Please provide p values in addition to 95% CIs in the main text and tables**

Authors’ reply: OK, p-values added in the results.

**12) Your study is observational and therefore causality cannot be inferred. Throughout the text, please remove language that implies causality, such as “impact” and “effects.” Refer to associations instead.**

Authors’ reply: Revised.

**13) Please define the abbreviations in Tables and Figures e.g, OR, IRR, CI, cons, FP, URTI, LRTI, ILI, ANC1**

Authors’ reply: OK, abbreviations clarified under each table and figure as needed.

**14) In figure 2 and parts of figure 3, please show the axis beginning at zero for both graphs. If this is not possible, please show a break in the axis.**

Authors’ reply: Thank you, the plot has been updated to start the y-axis at zero.

**15) Please do not report P<0.0001; report as P < 0.001.**

Authors’ reply: Noted, edited.

**16) Please remove the “Role of the funding source” statement in the methods section. This information is captured in the metadata obtained in the submission form**

Authors’ reply: Noted, removed.

**17) Please replace the term “compliance” with “adherence” where it is used to refer to treatment adherence.**

Authors’ reply: Noted, edited.

**18) In the conclusion of the discussion, please present implications and next steps for research, clinical practice, and/or public policy; one-paragraph conclusion.**

Authors’ reply: We added a one-paragraph conclusion (page 25).

**19) Please remove the “Competing Interests”, “Data availability,” and “Funding” statements at the end of the main text. This information is captured in the metadata obtained in the submission form.**

Authors’ reply: Noted, removed.

**20) Please move “Ethics Approval” to the Methods section.**

Authors’ reply: Noted, moved.

**21) References:**

a) **Please select the PLOS Medicine reference style in your citation manager. In-text reference call outs should be presented as follows noting the absence of spaces within the square brackets, e.g., "... services [1,2]."**
b) **References should have no less and no more than six names before et al. For those with more than six names, please ensure that et al., is inserted after six names**
c) **Please ensure that journal name abbreviations consistently match those found in the National Center for Biotechnology Information (NCBI) databases.**

<https://nam02.safelinks.protection.outlook.com/?url=https%3A%2F%2Fjournals.plos.org%2Fplosmedicine%2Fs%2Fsubmission-guidelines%23loc-references&;data=04%7C01%7Cpbspiegel%40jhu.edu%7Cc11a229c41f94ee4cf3008d9fd541086%7C9fa4f438b1e6473b803f86f8aedf0dec%7C0%7C0%7C637819359772374136%7CUnknown%7CTWFpbGZsb3d8eyJWIjoiMC4wLjAwMDAiLCJQIjoiV2luMzIiLCJBTiI6Ik1haWwiLCJXVCI6Mn0%3D%7C3000&sdata=%2FSSHNkZJbFsED2hqbMr%2BXcjalFWoM21dJ4D%2BJJgSAEc%3D&reserved=0>.

Authors’ reply: Noted, Vancouver style with brackets and max 6 names before et al was used for references.

**Comments from the reviewers:**

**Reviewer #1:**

Dear Authors,

Thanks a lot for this very interesting and informative article. It contains such an important information in the care of refugees in the humanitarian setting, and should be published.

I have three comments (and inquiries).

1.3 **First is the covid-19 diagnostic process and treatment. I assume Syrian refugees in both camps have full and free access to covid-19 diagnosis (PCR) and treatment (hospitalization when needed). I suggest elaborating this, if not yet, in the manuscript.** **When such access is not free nor easy, people may behave differently: i.e., may not seek PCR tests. I am raising this to exclude the possibility that lower incidence rate is not the product of limited access to PCR & treatment. For Jordanians, it’s free in both PCR tests and hospitalizations at government facilities. Is this the same for Syrian refugees in both camps?**

**If PCR & hospitalization access is free for Syrian refugees, I would appreciate if you could mention this, with possible appraisal, in the manuscript. As for Palestine refugees (PR) we work for, they included all PR in their covid-19 care without discrimination. This is actually not common in humanitarian setting.**

Authors’ reply: Thanks, this is an important point. Refugees had access to free testing and treatment, as nationals. We revised the discussion accordingly on pages 21 and 22.

**1.2 Second is the political / security risks of covid-19 infection among Syrian refugees in the camps. In some settings, covid-19 infection would negatively affect their livelihood access: e.g., work permission or movement permission. If there are such cases, people may access PCR less with the fear of losing work or movement permissions. Is there any situation like this for Syrian refugees in the camps?**

Authors’ reply: Thanks, this is another important point. We addressed both this and the previous point in the discussion (pages 21 and 22).

**1.3 Third is vaccinations.** **This would surely affect the severity of the covid-19 infection. If not yet, possible for you to address this in our manuscript? Again, Jordan government included refugees (at least Palestine refugees I know the best) in their national deployment and vaccination programs. This itself is a great act. Is it the same for Syrian refugees in the camps?**

**Again, thanks so much for your very important manuscript.**

Authors’ reply: Thank you. We recognize vaccination is an important issue. As the study covers the period March 2020 to March 2021, however, we cannot include vaccination in this analysis. We have however added reference to vaccination in the discussion. It is an important issue to be addressed in future studies.

**Reviewer #2:**

**Altare et al y describe the epidemiology of COVID-19 in Azraq and Zaatari refugee camps (Jordan) and evaluate its impact on routine health services. Cases were first reported six months after the first case was reported in Jordan. Incidence rates were lower in camps than neighboring governorates and the country as a whole. Characteristics of cases and risk factors for negative disease outcomes were consistent with evidence from elsewhere. Overall health care utilization, consultations for respiratory infections, immunization and family planning declined during the first year of COVID-19, while maternal health services and non-communicable diseases were less affected. The authors report that COVID-19 transmission was lower in camps than outside of camps. Health services were affected differently, but disruptions appear to have been limited.**

**2.1 This is a well written and well analyzed description and the authors should be commended for their clarity and comprehensive analyses. I do not have any comments on this piece other than one key aspect. Although the authors mention, in the discussion (in fact only once on page 18 -"These results support the assertion that refugee populations living in the two largest camps in Jordan did not represent a threat of spreading SARS-COV-2 amongst the general population, as has been claimed in many countries hosting refugees" - this is in fact major in terms of the report presented here. The manuscript could be enhanced by providing additional information on this aspect as well as additional commentary from the authors. The "blaming" of refugees is both common and often egregious. I would strongly suggest to the authors to extend this discussion more and even consider being a bit stronger in the abstract, highlighting this point or consider adding a concluding paragraph to the discussion.** **Other than that, there are some minor typos which the authors can be ensured to address in the text.**

Authors’ reply: Thank you. We agree with your comment and expanded the discussion on page 22.

**Reviewer #3:**

Comments are in the attached document. And pasted here after:

**3.1 The article bares very little comments. It is comprehensive, quite unique in its focus, is well documented and referenced, with a rich set of conclusions which interestingly are not necessarily the intuitive ones readers would expect.**

Authors’ reply: Thank you.

**3.2 Authors should therefore be praised for this detailed work in collecting and processing this innovative set of evidence on refugee camps in times of COVID-19**

Authors’ reply: Thank you.

**3.3 The article may however provide a misperception about refugees in closed camps setting as if such camps conditions constitute in themselves "counter-measures" for pandemic spread. The various biases and limitations in the interpretation of the data set are very well explained, but a reminder that the situation in these camps should not be seen as pandemic mitigation factors and generalized as such. It is obvious that this would not be the intention of the authors, but it could be a side effect of the way these conclusions are presented - without cautioning statement. A sentence or two should therefore warn against any arguments equating refugee protection condition to refugee health. It is Jordan's and UNHCR merit to ensure universal access to adequate health services to refugees in these camps, but these are still 'camps' with living conditions marked by severely restricted liberties to be no justification for protection against COVID-19.**

Authors’ reply: This is an important point, and thank you for raising it. Indeed, it was not our intent to present camp-like setting as a desirable solution to prevent COVID. We edited the discussion accordingly.

**3.4 Although vaccines were not available at the time of the study, the conclusions of COVID-19 being a milder problem (than expected) in refugee camp settings may be misused politically to delay access to vaccines for refugees nowadays. This may be off-subject here, but a sentence should highlight that - given the parallel in this data set with what we know of COVID-19 -vaccination is likely to hold the same benefit in refugee camps than anywhere else, and that refugee camps should remain a top priority for any national COVID-19 vaccination roll-out.**

Authors’ reply: We do agree with this sentiment. However, since we did not address vaccinations in this article, we prefer not to mention it in the discussion.

3.5 **The impact of NPIs in camp setting, which can be implemented more easily than in open societies, is well mentioned, but would deserve more elaboration: it could be good to have (if possible) them - if not quantified (stringency index overtime) - at least better qualified, so that we can have a better understanding of their greater impact in closed camps than outside them.**

Authors’ reply: A stringency index like the Oxford one was not calculated for camps and we are not able to add a quantification of the NPI. However, we have added a table in supporting material that describe the measures that were introduced in camps.

3.6 Finally, **the favorable course of the pandemic in these two camps compared to the outside communities in Jordan cannot be replicated to migrant camp settings such in Greece. This is evidently another subject, but in the commentary section, a couple of sentences contrasting the two situations could be added.** **Reference to: S Hargreaves, E Kondilis, D Papamichail, S McCann, M Orcutt, E Carruthers, A Veizis, The impact of COVID-19 on migrants in Greece: a retrospective analysis of national data, European Journal of Public Health, Volume 31, Issue Supplement_3, October 2021, ckab164.589,** [**https://doi.org/10.1093/eurpub/ckab164.589**](https://doi.org/10.1093/eurpub/ckab164.589)**.**

**Again, the intention is to avoid overinterpreting these conclusions to setting that may overlap but which bare very different assumptions in terms f protection, level of care, and state oversight.**

Authors’ reply: Thanks for raising this important point. We agree with you and have revised the discussion accordingly (page 25).

**3.7 To mention a typo in the last sentence: " there are no available data are available to analyze its effects "**

Authors’ reply: Noted, thank you. It has been corrected.

**3.8 In conclusion: a great article with important data and new evidence presented, deserving immediate publication without revisions, apart from cautioning against possible misuse or over/misinterpretation of some of the conclusions at political level.**

Authors’ reply: Thank you.

**Reviewer #4:**

**1. The title of the article does not fully reflect that one of the primary objectives is to assess the impact on routine health service utilization.**

Authors’ reply: Agreed. A new title has been suggested (see above).

**2. As model equation on page 4 indicates the author used a generalized linear model, what's the reason to apply the generalized additive model using mgcv function,  which is usually used to capture non linearities by adding non-linear smooth functions, rather than the generalized linear model using package such as glm.nb()?**

Authors’ reply: Thank you for this question. Indeed, for this analysis, we did not include any smooth terms, so the model is identical to a generalized linear model using glm.nb() from the MASS package. Prior to deciding on this model specification, we tested other more flexible models that did include smooth terms, but for the large part, did not see much improvement. Hence, we retained the generalized additive model framework for convenience, while recognizing that without the smoother terms, the model is functionally equivalent to a generalized linear model. To double check, we verified that the results are the same by running the same analysis for the overall consultations indicator, and confirmed that the output is the same. We added a sentence in the *Statistical analysis* sub-section of *Methods* to acknowledge this point.

**3. Since the outcome of interest is incidence/utilization rate, I would suggest to use offset(log(population)) instead of ß1 log(population).**

Authors’ reply: Thank you. We agree with this point. Analysis had been rerun using offset(log(population)) instead, and the model equation in the *Methods* section has been updated to reflect this change. Results remained substantially unchanged except for mortality (now significant in Azraq), all respiratory infections (now all significant), and measles (now with a pvalue of 0.06). Results have been updated accordingly.

**4. Are the ORs reported in Table adjusted or unadjusted? Are seasonaly pattern and secular trend taken into consideration?**

Authors’ reply: Odds ratios for COVID outcomes are adjusted. Caption of table 2 has been edited for clarity.

**5. Page 10:  "Azraq recorded an immediate decrease of 30% (IRR: 0.705, 95%CI [0.533 - 0.933]) and Zaatari of 21% (IRR: 0.794, 95%CI [0.597 - 1.056]).". Was the 30% decrease significantly different from the 21% decrease in Zaatari? The author may need to test this by using interaction.**

Authors’ reply: Thank you for this question. We constructed the model separately for each of the two camps, and did not explore whether the *differences* between the camps were statistically significant. From operational standpoint, given that the populations across the two camps, implemented programs and partners are different, we did not explore whether the changes during COVID-19 were different across the two camps, but focused on understanding what happened in each camp.

**6. Figure 2 indicates the recovery of health utilization since the immediate decrease of 30% in outpatient visits in Azraq in Jan 2020, while no evidence of recovery in Zaatari. I would suggest to test it and present the results.**

Authors’ reply: Thank you for this suggestion. As in the comment above, we prefer to limit this study to understanding what happened within each camp without directly comparing the two.

**7. How did the author deal with potential outliers?  Particularly in the subgroup analysis for ANC coverage, lie birth coverage and measles vaccination.**

Authors’ reply: Thank you, we added a short explanation in the *Methods* section. Across all indicators, we defined potential outliers as any values in the pre-COVID-19 period that were at least 3 standard deviations away from the mean. We removed these outliers prior to carrying out analysis. For measles vaccination, we did remove a major spike in mid-2018, which was due to a supplementary immunization campaign carried out in the two camps.
