# Supplementary material for: COVID-19 epidemiology and changes in health service utilization in Azraq and Zaatari refugee camps in Jordan: A retrospective cohort study
Source: PLoS Med. 2022 May 10;19(5):e1003993. doi: 10.1371/journal.pmed.1003993 (PMC9089859; doi:10.1371/journal.pmed.1003993)
Supplement: Attachment — Submitted filename: Responses to comments_04162022.docx [file pmed.1003993.s007.docx]

**Comments to the article: COVID-19 pandemic in Azraq and Zaatari refugee camps in Jordan: are refugees in camps at reduced risk?"**

**(PMEDICINE-D-22-00341R1) for consideration at PLOS Medicine.**

Baltimore, April 14, 2022

Dear Editors and reviewers,

Thank you for accepting our paper for publication.

Please find below the point-by-point responses from the authors to the remaining comments by the editors and reviewers.

We remain at your disposal for additional clarifications and next steps.

Best wishes,

Paul Spiegel

**Requests from Editors:**

**1. For the data availability statement - If the data are not freely available, please include an appropriate contact (web or email address) for inquiries (this cannot be a study author)**

Ok, we entered this revised data availability statement in the submission form:

Anonymized and de-identified individual-level data (COVID-19) and aggregated weekly data (routine health services) may be made available upon request following publication and approval of a concept note summarizing the analyses to be conducted. Please contact Johns Hopkins’ Center for Humanitarian Health at [humanithealth@jhu.edu](mailto:humanithealth@jhu.edu).

**2. Thank you for providing the STROBE checklist. Please upload the STROBE checklist as a separate supporting file (titled, “S1 Checklist”) and reference it accordingly in the Methods section.**

Noted. We uploaded two files S1 Checklist, and S2 Supplementary information.

Method section has been revised accordingly as well as references to S2 files were added where needed in results and discussion.

**3. The last sentence of the Introduction needs revision as causal effects cannot be inferred, from “…and evaluate the effects of COVID-19 and its related response measures…” to “… and evaluate the associations between COVID-19 and routine health service utilization…”**

Noted. We revised the sentence into “evaluate the changes in routine health service utilization..”.

**Comments from Reviewers:**

**Reviewer #1: Thank you so much. You kindly and nicely addressed the points i raised previously. Very clear and very well-explained!**

Thank you.

**Reviewer #2: The authors have responded to the questions posed by all reviewers. My recommendation would be to accept this manuscript for publication.**

Thank you.

**Reviewer #4: Thank you for improving the manuscript.**

**Just one more minor comment**

**1. The author may need to replace the term "OR (odds ratio) " by "IRR (Incidence rate ratio) " to make it consistent although they are often used interchangeably in the interpretation of the results from negative binomial models.**

Thanks for this comment – we would prefer keeping IRR for the results of the ITS, and OR for the logistic regression to reflect the way in which they have been calculated.
